# Reinforcement Learning of Chaotic Systems Control in Partially Observable Environments

**DOI:** 10.1007/s10494-024-00632-5

**Published:** 2025-01-09

**Authors:** Max Weissenbacher, Anastasia Borovykh, Georgios Rigas

**Affiliations:** 1 Department of Mathematics, Imperial College London, London, SW7 2AZ UK; 2 Department of Aeronautics, Imperial College London, London, SW7 2AZ UK

**Keywords:** Reinforcement learning, Active flow control, Chaos, Optimal control

## Abstract

Control of chaotic systems has far-reaching implications in engineering, including fluid-based energy and transport systems, among many other fields. In real-world applications, control algorithms typically operate only with partial information about the system (*partial observability*) due to limited sensing, which leads to sub-optimal performance when compared to the case where a controller has access to the full system state (*full observability*). While it is well-known that the effect of partial observability can be mediated by introducing a memory component, which allows the controller to keep track of the system’s partial state history, the effect of the type of memory on performance in chaotic regimes is poorly understood. In this study we investigate the use of reinforcement learning for controlling chaotic flows using only partial observations. We use the chaotic Kuramoto–Sivashinsky equation with a forcing term as a model system. In contrast to previous studies, we consider the flow in a variety of dynamic regimes, ranging from mildly to strongly chaotic. We evaluate the loss of performance as the number of sensors available to the controller decreases. We then compare two different frameworks to incorporate memory into the controller, one based on recurrent neural networks and another novel mechanism based on transformers. We demonstrate that the attention-based framework robustly outperforms the alternatives in a range of dynamic regimes. In particular, our method yields improved control in highly chaotic environments, suggesting that attention-based mechanisms may be better suited to the control of chaotic systems.

## Introduction

Chaotic systems are ubiquitous in science and engineering, manifesting in diverse fields from meteorology to fluid dynamics. These systems, characterized by their sensitivity to initial conditions and complex, unpredictable behaviour, play crucial roles in phenomena such as weather patterns (Lorenz 1963; Palmer 1993), turbulent flows in aerodynamics (Holmes 2012), and the intricate dynamics of superconductors (Blatter et al. 1994; Fisher et al. 1991). A critical challenge arises in controlling these chaotic systems, particularly in active flow control, where oftentimes the system state can only be partially observed due to engineering restrictions. Improvement of control in the partially observable setting could have significant implications in realistic engineering applications such as drag reduction in road vehicles, wind farm optimization, and enhanced chemical mixing processes (Rathnasingham and Breuer 2003), with potential contributions to global energy efficiency and net-zero emissions targets. This limitation in observational data leads to a decrease in controller performance (Kaelbling et al. 1998; Hausknecht and Stone 2015; Sondik 1978). Although it is well understood that incorporating a memory component into the controller can mitigate this issue, the question of which memory type best suits chaotic systems remains open. One compelling reason to investigate this issue is that recurrent neural networks, the standard approach for incorporating memory into controllers, are well known to suffer from error accumulation over time (Bengio et al. 1994; Pascanu 2013; Greff et al. 2016). This is a significant drawback for chaotic systems, where even small state deviations can lead to significant, unpredictable changes in behaviour. We address this gap by comparing a traditional LSTM-based memory with a novel attention-based memory method, demonstrating that the attention-based approach achieves superior performance in controlling chaotic dynamics.

In Sects. 1.1 through 1.4, we provide an overview of the use of reinforcement learning in active flow control, the issues arising with partial observability and discuss the model system used in this study. In Sect. 1.5 we highlight the contributions of this work to the field.

### Maximum-Entropy Reinforcement Learning

Active open-loop techniques in flow control (Brackston et al. 2016; Beaudoin et al. 2006; Giannenas et al. 2022), where pre-determined signals drive actuators, are often energy inefficient and lead to sub-optimal performance. This motivates the use of closed-loop (feedback) control. Reinforcement learning (RL) is a powerful data-driven framework for sequential decision-making, where an agent learns to make decisions by interacting with an environment in order to maximize a cumulative reward (Sutton and Barto 2018).

RL has been successfully used in a variety of active closed-loop flow control applications, and we will briefly highlight some selected works here. Rabault et al. (2019) and Rabault and Kuhnle (2019) applied RL to control the wake of a two-dimensional bluff body flow in a laminar regime. Recently, Xia et al. (2024) successfully demonstrated wake control of a two-dimensional bluff body flow in the periodic regime. Chatzimanolakis et al. (2024) explore the use of RL to control drag in flows past both 2D and 3D circular cylinders at Reynolds numbers up to $\text {Re}=8000$. They find that the control policies learned in 2D generalise well to the 3D scenario. Vasanth et al. (2024) and  Jeon et al. (2024) apply multi-agent RL to control 3D Rayleigh–Bénard convection outperforming the classical proportional control strategy for reducing convection. The study of optimal sensor placement contrasts with the problem of partial observability, where controllers work with suboptimal information. Paris et al. (2021) addresses optimal sensor placement in drag reduction for 2D laminar flow over a cylinder using synthetic jets, presenting a reinforcement learning approach that uses only five (optimal) sensors while maintaining top performance across varying Reynolds numbers and noisy conditions. For a more comprehensive overview of the use of RL in active flow control, we refer the reader to Vignon et al. (2023).

Maximum entropy RL algorithms address the challenges of learning optimal policies in environments with high-dimensional state spaces and complex dynamics by maximizing not only the expected cumulative reward but also the entropy of the policy, which encourages exploration and results in more robust policies. Soft Actor-Critic (SAC) (Haarnoja et al. 2018) is an off-policy algorithm that optimizes a stochastic policy in an entropy-regularized framework, leading to improved exploration and stability. By incorporating entropy into the objective, SAC promotes diverse action selection and improves the learning efficiency in complex environments. Truncated Mixture of Continuous Distributional Quantile Critics (TQC) (Kuznetsov et al. 2020) further builds upon SAC, extending its capabilities by introducing quantile regression to predict a distribution for the value function (instead of a mean value). TQC enhances robustness by estimating the entire distribution of potential future rewards.

### Partial Sensor Measurements and POMDPs

In realistic engineering applications, typically only partial observations are available to the controller. Consider the example of reducing the drag of a road vehicle for instance; in this case sensors will be located only on the vehicle’s body. In RL, the system dynamics are assumed to be described by a Markov Decision Process (MDP). In an MDP, the environment dynamics satisfy the Markov property (Dodge 2003), meaning that the probability of transitioning to a new state depends solely on the current state and action, independent of prior history. This property means that RL algorithms can reliably converge to an optimal policy under certain conditions, such as sufficient exploration and an appropriately tuned learning rate (Sutton and Barto 2018).

If only a limited number of sensors is available to the controller on the other hand, the environment is more accurately described by a Partially Observable Markov Decision Process (POMDP). The Markov property does not hold for POMDPs (Kaelbling et al. 1998; Cassandra et al. 1994). As a consequence, RL algorithms in POMDPs face significant challenges in convergence, as they require sophisticated methods to estimate the state and integrate it into decision-making, resulting in less predictable and often slower convergence behavior (Kaelbling et al. 1998; Hausknecht and Stone 2015; Sondik 1978). We present MDPs and POMDPs in more technical detail in Sect. 2.3.

### Reducing POMDPs to MDPs by adding Memory

In order to convert a POMDP into an MDP, the controller can be augmented with a ‘memory’, which keeps track of previous partial observations and is continually updated as new observations are made, allowing the controller to access past information. The problem of partial observability and the use of memory to improve controller performance is well-studied in the active flow control literature. Xia et al. (2024) study the problem of controlling a 2D bluff body flow at Reynolds number $\text {Re} = 100$ using only partial observations given by pressure sensors located at the back of the bluff body. They show that optimal performance can be recovered when using only partial observations of the flow field by incorporating a finite-time history of past observations (or delay embedding) into the controller, see Sect. 2.4 for a detailed discussion of this method. Wang et al. (2024) study the fluid flow around a 3*D* circular cylinder for Reynolds numbers ranging from $\text {Re} = 100$ to $\text {Re} = 10000$. They use a modified delay embedding of sparse surface pressure sensors to recover the performance of a controller with sensors in the wake, leading to significant improvements in drag reduction and lift fluctuation mitigation. In the present study, we focus on the comparison of different frameworks for incorporating memory in highly chaotic control environments.

In the RL literature, the pre-dominant method of endowing controllers with memory is the use of recurrent neural networks (RNNs), see for instance (Wijmans et al. 2023; Peng et al. 2018; Hausknecht and Stone 2015; Zhu et al. 2017). More recently, the concept of attention has been used to enable RL agents to solve POMDPs more effectively. Attention-based architectures, such as Transformers (Vaswani et al. 2017) originally emerged in the context of language modelling and have proven to be highly effective in this domain. The Transformer architecture has significantly improved natural language understanding and generation, leading to breakthroughs in machine translation (Devlin et al. 2018), text summarisation (Radford et al. 2019), and conversational AI (Brown et al. 2020). Beyond language processing, Transformers have also been adapted for tasks in computer vision (Dosovitskiy et al. 2020), time series forecasting (Wen et al. 2022) and recently in reinforcement learning (Santoro et al. 2018; Parisotto et al. 2020; Pritzel et al. 2017).

In this work, we implement CHAROT, an attention-based memory framework by the authors, originally introduced in Weissenbacher et al. (2024). We then compare this framework to a memory-less controller and a controller augmented with an RNN.

### The Kuramoto–Sivashinsky Equation and Phases of Chaos

In our work we study control of the Kuramoto–Sivashinksy (KS) equation (Kuramoto 1978; Ashinsky 1988; Sivashinsky 1980) in a chaotic regime. The KS equation models a range of physical phenomena, such as chemical reaction-diffusion systems (Kuramoto and Tsuzuki 1976) and laminar flame front instabilities (Ashinsky 1988). While the KS equation does not directly model hydrodynamic turbulence, it shares certain features with the Navier–Stokes equation and is often studied as a simplified toy model for chaotic dynamics in this context (Dankowicz et al. 1996). The dynamics of the equation depend on the value of a parameter $\nu$ (or equivalently, the length of the domain on which solutions are defined) and range from periodicity, oscillatory behavior, to high-dimensional chaos.

Existing studies on RL applied to the KS equation use the full system state as a controller input (full observability) and have mostly been limited to the periodic or quasi-periodic regimes. In fact, the existing literature on RL applied to the KS equation considers the KS environment on a domain of length $L=22$, or equivalently $\nu \approx 0.08$ (rounded). For this value of $\nu$, the dynamics of the system are confined to oscillations between a set of six steady state and travelling wave solutions and can therefore not be considered to be truly chaotic. Bucci et al. (2019) apply the Deep Deterministic Policy Gradient (DDPG) algorithm (Lillicrap et al. 2015) to steer the KS environment from one non-trivial steady state solution to another. Zeng et al. (2022) combine a reduced-order model with DDPG to achieve accelerated convergence. Zeng and Graham (2021) employ symmetry reduction methods combined with DDPG and establish that performance is improved by exploiting the symmetries of the KS equation. Xu and Zhang (2023) study control of the linearised KS equation, which may be interpreted as describing convectively unstable flat-plate boundary layer flow in the vicinity of the boundary.

In this work, we study the KS equation in highly chaotic regimes, where the parameter $\nu$ satisfies $\nu \ll 1$, so that dynamics are fully chaotic. This increases the effective dimension of the control problem compared to the case where $\nu \approx 0.08$, see the discussion in Sect. 2.2.

### Contribution

In the present work, we use RL to control highly chaotic flows under the restriction of partial observability. First, we quantify the loss of performance as the number of sensors available to the controller decreases and the control problem transitions from being fully observable to partially observable. Second, we propose a novel way of reducing the POMDP to an MDP by using an attention-based memory framework, called CHAROT. We compare our novel method to the established method of using an RNN-based memory for reducing the POMDP to an MDP. We demonstrate that our method robustly outperforms the classical RNN-based memory framework. The main contribution of our work is to demonstrate that attention-based methods are more effective than traditional RNN-based approaches for integrating memory into RL policies, particularly in scenarios with partial observability. This finding highlights the superior capability of attention mechanisms to handle complex dependencies and retain relevant information in such environments.

### Overview

In Sect. 2, we introduce the KS environment and discuss the dynamics of the KS equation in some detail. We also discuss the theoretical framework behind POMDPs and introduce the three different RL frameworks which are being compared in this study. In Sect. 3 we present the results of our investigations, including details on the training process. In particular, we quantify the loss of performance of a memory-less controller as the number of sensors decreases, we compare the memory-less controller to two memory-based frameworks, and we assess how well each of the frameworks generalise to different regimes of chaos. We provide conclusions for the current research and future research directions in Sect. 4.

## Methodology

We compare three RL controller frameworks on the KS environment in different dynamic regimes. The RL agent controls a forcing term in the equation and aims to stabilise the system around a fixed point solution. The agent is only able to partially sense the environment at any given time.

We introduce the environment in Sect. 2.1. The dynamics of the unforced KS system are complex and relevant to our study. We discuss the dynamics of the unforced KS system in Sect. 2.2. In the following Sect. 2.3 we touch upon the theoretical framework underpinning RL with partial observations. Finally, we describe in some detail the three different RL frameworks used in this study in Sect. 2.4.

### The Kuramoto–Sivashinksy Environment

The environment is a numerical simulation of the Kuramoto–Sivashinksy (KS) equation, with an inhomogeneous forcing term to introduce control to the problem. The KS equation is the fourth-order nonlinear partial differential equation given by

1 $$\begin{aligned} \mathcal {L}(h) = \partial _t h + \partial _{xx} h + \nu \partial _{xxxx} h + \frac{1}{2} (\partial _x h)^2 = f, \end{aligned}$$

where $h: [0, \infty ) \times [0,2\pi ] \rightarrow \mathbb {R}$ satisfies $h(t, 0) = h(t, 2 \pi )$ for all $t \ge 0$ (periodic boundary conditions) and *f* is a forcing term. The parameter $\nu > 0$ plays an important role in the dynamics of the system. Often, the equation is instead given in the form of (1) where the parameter $\nu$ is set to $\nu =1$ and the equation is instead solved on the periodic domain [0, *L*] for some $L>0$. The two formulations are entirely equivalent if $L = 2 \pi \nu ^{-\frac{1}{2}}$, as may be seen from a simple scaling argument.

The Control Problem: Gaussian Actuators To turn this into a control problem, we introduce an in-homogeneous forcing term. Let *A* be the number of actuators and let $x_1, \dots x_A \in [0, 2 \pi ]$ be equi-spaced points in the domain. We then define the controlled system

2 $$\begin{aligned} \mathcal {L}(h) = f(\vec {a}), \quad f(\vec {a}; x) = \sum _{i=1}^A a_i e^{-\frac{1}{2}\left( \frac{x - x_i}{\sigma }\right) ^2}, \end{aligned}$$

where $\sigma >0$ describes the width of each Gaussian actuator. The mixture weights $\vec {a} = (a_1, \dots , a_A)$ are dynamically determined by the controller. In order to define an optimal control problem, we introduce the cost functional (or energy)

3 $$\begin{aligned} \mathcal {E}(h) = \frac{1}{T} \int _0^T \int _{0}^{2\pi } \left| h(t, x) \right| ^2 \, dx dt = \frac{1}{T} \Vert h \Vert _{L^1_t L^2_x}, \end{aligned}$$

where $T > 0$ denotes the length of an episode. The initial condition $h(0,x) = h_0(x)$ are sampled to be a Gaussian random perturbation of the zero solution. The cost functional is minimised when $h = 0$. Hence, minimising the cost functional $\mathcal {E}$ starting from initial data which are a random perturbation of the zero solution amounts to stabilising the system around the zero solution.

For each parameter $\nu < 1$, the homogeneous KS equation possesses finitely many steady-state solutions. The constant zero solution $h = 0$ is the most unstable with $\lfloor 2 \nu ^{- \frac{1}{2}} \rfloor$ unstable real modes, where $\lfloor x \rfloor$ is the largest natural number *n* such that $n \le x$. Therefore, we may conclude that the zero solution is the most challenging solution to stabilise.

Numerical Methods We solve the KS equation using a third-order Runge–Kutta scheme. We use a timestep of $dt = 0.05$ to propagate the equation forward in time and chose $N_F=64$ Fourier modes, which is sufficient to resolve the system for the range of $\nu$ considered in this study ($5 \times 10^{-3} \le \nu \le 5 \times 10^{-2}$). The Cauchy problem for the KS equation is well-posed for periodic initial data $h(0,x) = h_0(x)$ (Tadmor 1986). Moreover, the KS equation possesses a smoothing property similar to the heat equation: under some mild regularity assumptions, periodic initial data give rise to solutions which are real analytic (Collet et al. 1993). As suggested by the presence of chaotic solutions, the KS equation is non-integrable and no closed-form explicit solutions exist (Conte and Musette 1989).

The continuous cost functional $\mathcal {E}$ (or energy) is numerically approximated by

4 $$\begin{aligned} E = \frac{1}{N} \sum _{n=0}^N \int _{0}^{2\pi } \left| h_n(x) \right| ^2 \, dx, \end{aligned}$$

where $h_n(x) = h(t_n, x)$ is the numerical solution at the discrete timestep $t_n = n \, dt$, $N=\frac{T}{dt}$ and the spatial integral is computed using the trapezoidal rule. We also define the *reward signal*

5 $$\begin{aligned} r_n = - \int _{0}^{2\pi } \left| h_n(x) \right| ^2 \, dx , \end{aligned}$$

so that $\frac{1}{N} \sum _{n=1}^N r_n = - E$. Notice the change of sign; this is a convention in the RL literature, where by convention the cumulative reward is typically maximised, while we are interested in minimising the energy functional.

Partial Observability The controller has access only to partial measurements of the solution $h_n = h(t_n, \cdot )$ from a limited number *S* of point sensors in the domain. Let $\bar{x}_1, \dots \bar{x}_S \in [0, 2 \pi ]$ denote *S* equi-spaced points and let

6 $$\begin{aligned} o_n = (h_n(\bar{x}_1), \dots , h_n(\bar{x}_S)) \end{aligned}$$

be the vector consisting of $h_n$ evaluated at the sensor locations. Instead of the full state $h_n$ (the numerical solution to the KS system at time $t_n = n \, dt$), the controller only sees the partial observation $o_n$.

**Fig. 1:**
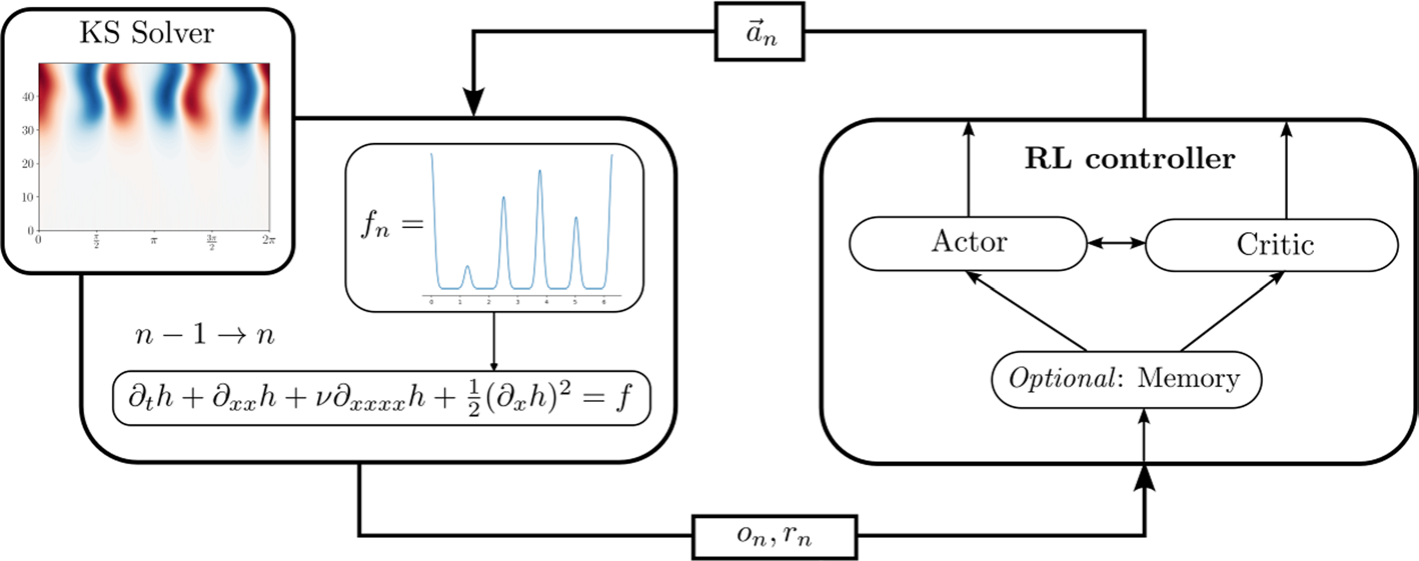
Overview of the RL feedback loop. The state of the KS environment (depicted on the left-hand side) gets updated using a numerical solver and the output from the RL controller, the Gaussian mixture weights $\vec {a}_n$. During training, the controller then receives the partial observation $o_n$ as well as the reward $r_n$. The controller optionally includes a memory framework to compensate for the lack of observations, see Sect. 2.4

Our setup closely mirrors a typical real-world flow control application. In realistic engineering applications, only a small discrete number of sensors is available. This resembles our problem setting: a highly chaotic flow which can only be sensed at a limited number of points in the flow domain and which must be steered towards an unstable (laminar) flow state.

In RL, it is common to use an ensemble of parallel simulation environments. Leveraging parallel simulated environments accelerates the learning process by providing diverse experiences to the agent, which facilitates better exploration, reduces the variance of gradient estimates and improves policy robustness (Mnih et al. 2016; Horgan et al. 2018; Espeholt et al. 2018; Heess et al. 2015). However, in a practical experiment or real-world engineering application, one typically only has one parallel environment. For this reason we choose to train our agents with only one parallel environment.

### Dynamics of the Uncontrolled KS Equation

The dynamics of the KS equation depend on the parameter $\nu$. While for $\nu \ge 1$, the trivial zero solution is stable, the KS equation exhibits complex spatio-temporal dynamics for $\nu < 1$. As $\nu$ decreases, the trivial zero solution becomes unstable to periodic solutions, traveling wave solutions, oscillatory solutions and eventually chaotic solutions (Cvitanović et al. 2010; Kalogirou et al. 2015). Solutions are typically considered to be fully chaotic from about $\nu \le 0.01$, for larger $\nu$ the periodic boundary conditions constrain the dynamics and ‘simple’ attractors exist (Wittenberg and Holmes 1999).

**Fig. 2:**
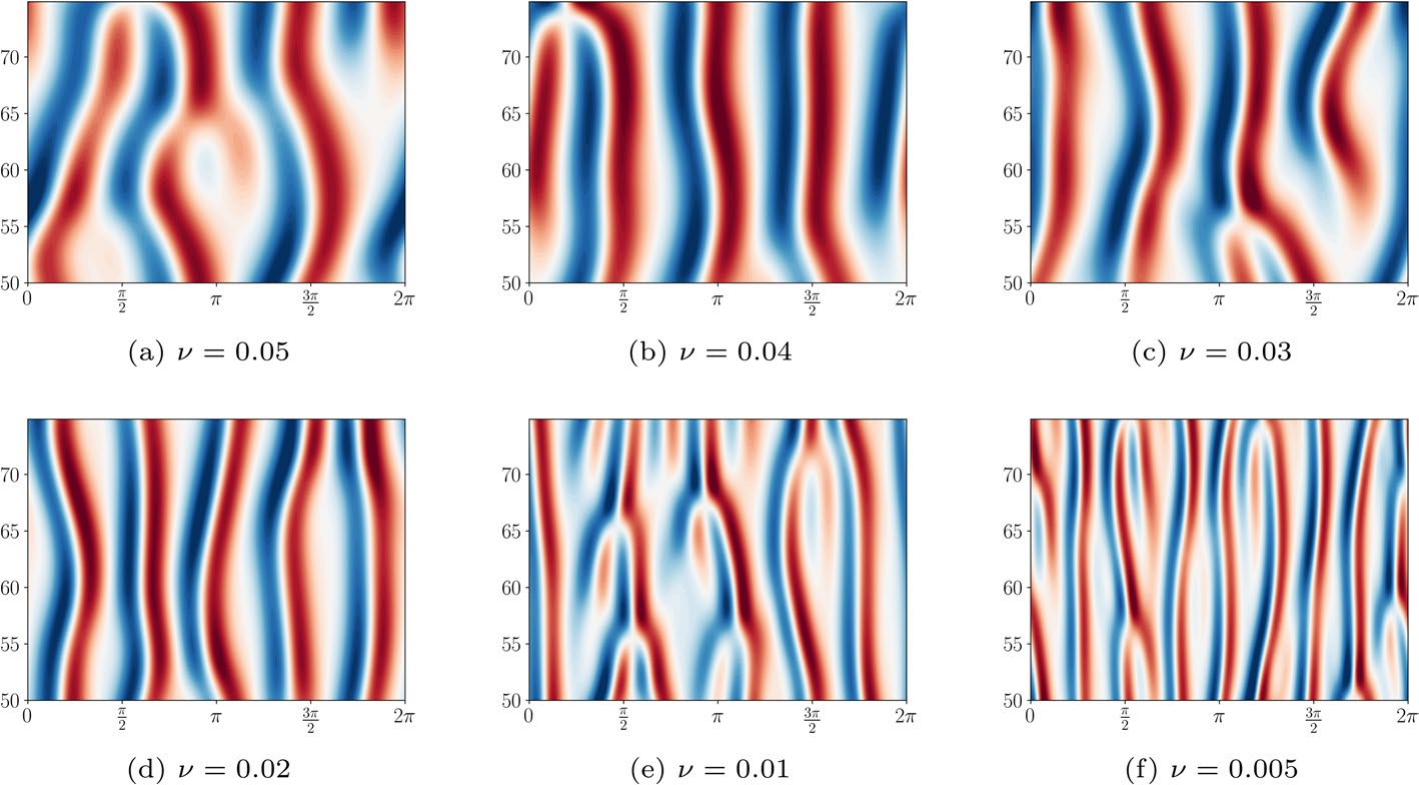
(*x*, *t*)-plots of uncontrolled solutions to the KS equation for decreasing values of $\nu$. The vertical axis is the time dimension. The solutions were obtained by selecting a random perturbation of the zero solution and solving for 500 discrete timesteps. Displayed are the solutions *h*(*t*, *x*) where $50 \le t \le 75$ and $x \in [0,2\pi ]$. The burn-in time of $t=50$ was selected so that all solutions converge to the chaotic attractor

**Fig. 3:**
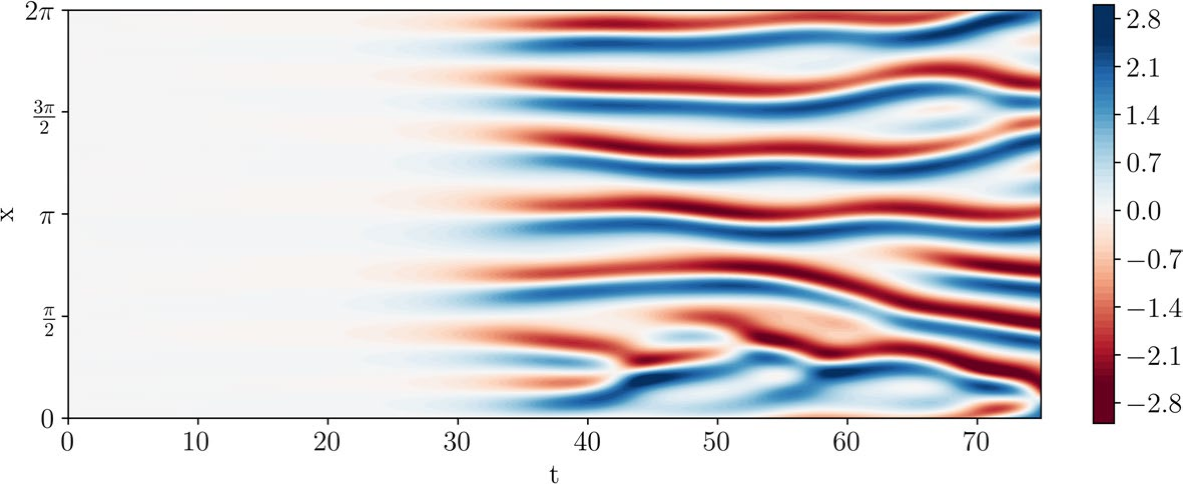
(t,x)-plot of an uncontrolled solution to the KS equation with $\nu =0.01$ and initial data chosen to be a random perturbation of the zero solution. The vertical axis is the spatial dimension. Note carefully that the solution converges to the attractor around $t \approx 30$. This justifies our choice of using episodes of length $T=50$ during training

Temam and Wang (1994) showed that all solutions of the KS equation approach a finite-dimensional inertial manifold. They established that the dimension of this sub-manifold is bounded from above by a constant times $(\log L)^{0.2} L^{1.64}$, where $L= 2 \pi \nu ^{-\frac{1}{2}}$. Figure 2 displays solutions converged to the inertial manifold for several values of $\nu$, while Fig. 3 shows the evolution of a solution from time $t=0$ onwards.

The inertial manifold contains an attractor which is conjectured to be fractal (Temam 2012). The Lyapunov dimension (Kaplan and Yorke 2006) is a measure of the fractal dimension of the chaotic attractor of a dynamical system. It is defined as a function of the Lyapunov exponents of the system and is computationally more tractable than the Hausdorff dimension. The Lyapunov dimension of the KS equation is conjectured to grow linearly with $L = 2 \pi \nu ^{- \frac{1}{2}}$, a claim which is supported by numerical studies (Collet et al. 1993; Edson et al. 2019), but has not been proven rigorously at the time of writing.

Table 1 lists the Lyapunov dimension and number of unstable modes of the trivial zero solution for a variety of values of $\nu$, as computed in Edson et al. (2019). Note that the Lyapunov dimension is not well-defined (or meaningful) for $\nu \ge 0.04$, since the system is not considered to be chaotic in this range. Pathak et al. (2017) provide a fast machine learning-based algorithm to approximate the Lyapunov times (and hence Lyapunov dimension) of the KS equation.

We also point the reader to further literature which analyses the bifurcation behaviour and long-term dynamics of the KS equation, see for instance (Nicolaenko et al. 1985; Papageorgiou and Smyrlis 1991; Linot and Graham 2020; Özalp et al. 2023). For the vast literature on analytical results for the KS equation, see for instance (Giacomelli and Otto 2005; Otto 2009; Foias et al. 1986).

Previous works studying control of the KS equation using RL limit their investigation on the KS environment to the case $L=22$, which in our formalism corresponds to $\nu \approx 0.08$, see also the discussion in Sect. 1. The KS equation is not fully chaotic for this choice, but in an intermediate regime between periodic and fully chaotic. Cvitanović et al. (2010) provide an in-depth study of the structure and dynamics of the attractor for the case $L=22$. The authors would like to emphasize that studying the KS environment for a larger range of values of $\nu$ reveals different qualitative dynamics and allows one to define the ‘hardness’ of the control problem.

**Table 1:** Lyapunov dimensions and number of linearly unstable modes of the zero solution for various values of the parameter $\nu$. The second column contains the equivalent domain length calculated as $L = 2 \pi \nu ^{-\frac{1}{2}}$ rounded to the nearest integer

$\nu$	*L*	Lyapunov dimension	Number of unstable modes
0.005	89	20	14
0.01	62	14	10
0.02	44	10	7
0.03	36	8	5
0.04	31	–	5
0.05	28	–	4
0.08	22	–	3

### Reinforcement Learning in Partial Observability

In reinforcement learning, the environment is formally modeled as a Markov Decision Process (MDP) (Sutton and Barto 2018), defined by the tuple $(\mathcal {S}, \mathcal {A}, \mathcal {P}, \mathcal {R})$. Here, $\mathcal {S}$ is the set of states representing the possible configurations of the environment, $\mathcal {A}$ is the set of actions available to the agent, $\mathcal {P}$ is the state transition probability function, $\mathcal {P}(s' \mid s, a)$, which defines the probability of transitioning to state $s'$ from state *s* after taking action *a* and finally $\mathcal {R}$ is the reward function, $\mathcal {R}(s, a)$, which specifies the immediate reward received after taking action *a* in state *s*.

A policy $\pi : \mathcal {S} \rightarrow \mathcal {A}$ is a mapping from states to actions. More generally, a probabilistic policy is a distribution $\pi (a \mid s)$ allowing to sample an action conditional on a state. Given a policy, we may define a rollout of the environment by the recursion relation

7 $$\begin{aligned} a_n&\sim \pi (\cdot \mid o_n ), \end{aligned}$$

8 $$\begin{aligned} r_{n}&= \mathcal {R}(o_n, a_n), \end{aligned}$$

9 $$\begin{aligned} o_{n+1}&\sim \mathcal {P}( \cdot \mid o_n, a_n). \end{aligned}$$

We then define the return of a policy as

10 $$\begin{aligned} R = \sum _{n=0}^\infty \gamma ^n r_n, \end{aligned}$$

where $0 < \gamma \le 1$ is the discount factor. A typical RL algorithm then seeks to find an optimal policy $\pi$ which maximizes the expected return.

We note carefully that if we define the reward $r_n$ as in eq.  (5), then the undiscounted return, as given by eq. (10) with $\gamma = 1$ satisfies $R = - N E$, where the discretised energy functional is defined in eq. (4). Therefore, maximising the reward amounts the minimising the cost functional. In practice, a discount factor $\gamma < 1$ is used, since this is found to lead to more robust convergence and stability.

In the case of the KS environment described in Sect. 2.1, the agent cannot directly observe the complete state of the environment. Instead, it receives partial observations $o_t$, which make up only a subset of the full state. This scenario is better described by a Partially Observable Markov Decision Process (POMDP), defined by the tuple $(\mathcal {S}, \mathcal {A}, \mathcal {P}, \mathcal {R}, \mathcal {O}, \mathcal {Z})$. Here, $\mathcal {S}, \mathcal {A}, \mathcal {P}, \mathcal {R}$ are defined as for an MDP and in addition $\mathcal {O}$ is the set of observations the agent can receive and $\mathcal {Z}$ is the observation function, $\mathcal {Z}(o | s, a)$, which defines the probability of receiving observation *o* given the current state *s* and action *a*.

### LSTM-Based and Attention-Based Memory

To convert the POMDP into an MDP, we augment the controller with a ‘memory’, allowing the controller to make use of past information to infer the state of the system.

Before discussing methods of incorporating past observations, we highlight an important design choice: the controller input may include or exclude the previous action taken by the controller. Ni et al. (2021) show that this choice can have a major impact on performance, but that this is highly dependent on the specific environment under consideration. In our study, early experiments quickly showed that performance of the controller did not improve when including the past action, so that we chose not to include it.

We also highlight here the choice of an actor-critic RL architecture. In our experiments, the base RL algorithm used is TQC. When augmenting the controller input, we may choose to augment only the input of the actor network, only the input of the critic network, or both. In our experiments, we chose to augment both the actor and critic, providing maximal information to both networks, see also Fig. 1.

Finite observation history The most direct method of incorporating past observations is to augment the controller input with a ‘finite history sufficient statistic’ Yu and Bertsekas (2008), a technique also known as ‘frame stacking’ in the context of RL (Mnih et al. 2013). The controller then takes the form

11 $$\begin{aligned} \pi (\vec {a}_n \mid o_{n}, \dots , o_{n-k}), \end{aligned}$$

where $k > 0$ is a natural number which describes the number of past observations (and actions, if included) incorporated into the controller. The theoretical justification for this method is grounded in Takens’s theorem Takens (2006), which states that the full state of a chaotic dynamical system may be reconstructed from the sequence of *k* past partial observations, assuming $k>0$ is large enough. We do not use frame stacking in this work, since it is known that recurrent neural networks reliably outperform this method, see the discussion in the following paragraph. We note here that a practical implementation of frame stacking typically involves finding an optimal number of past observations, however, we shall not discuss such issues further here since we do not make use of frame stacking in this work.

Recurrent neural networks An alternative method for incorporating past information into the controller is to augment the controller with a recurrent neural network (RNN), see (Yu et al. 2019) for an overview on RNNs. An RNN updates a hidden state $H_n$ using the new observations $o_n$ in a recursive (or sequential) manner:

12 $$\begin{aligned} H_n = \text {RNN}(H_{n-1}, o_n). \end{aligned}$$

The controller then takes the form

13 $$\begin{aligned} \pi (\vec {a}_n \mid H_n, o_n), \end{aligned}$$

where $H_n$ is updated by the RNN according to (12). Note that the controller is conditioned on the state $o_n$ even though it is also used as input to the RNN. This is an implementation detail that is often found to lead to better performance in practice, by allowing the controller to make use of the most recent observation in a more effective way.

Morad et al. (2023) evaluate frame stacking and numerous recurrent architectures on a comprehensive benchmark of partially observable game-like and abstract diagnostic environments. They find that the GRU (Dey and Salem 2017) and LSTM (Hochreiter and Schmidhuber 1997) architectures are among the best performing RNNs on their benchmark and consistently outperform frame stacking. This is consistent with the prevalence of LSTMs and GRUs in the RL literature, see for instance (Wijmans et al. 2023; Peng et al. 2018). In our study, we therefore choose to augment our controller with an LSTM as a baseline memory framework.

### Augmenting the controller with an attention-based memory

We now describe in detail our implementation of CHAROT  (**Cha**os **Ro**bust **T**ransformers), a novel attention-based memory framework which was originally introduced in our companion paper (Weissenbacher et al. 2024). CHAROT  is a drop-in replacement for the RNN in eqs.  (12, 13). The framework keeps track of a ‘memory’ $M_n$ and updates it recursively:

14 $$\begin{aligned} M_n = \text {CHAROT}(M_{n-1}, o_n). \end{aligned}$$

The memory $M_n$ is entirely analogous to the hidden state $H_n$ of an RNN. The controller then has a completely analogous structure to the RNN case,

15 $$\begin{aligned} \pi (\vec {a}_n \mid M_n, o_n), \end{aligned}$$

where the memory $M_n$ is updated according to (14). At its heart, the architecture consists of a modified Transformer with a slow-moving update.

The memory $M_n$ is a $n \times s$ matrix, where *n* is the number of memories and *s* is the size of each memory. Both *n*, *s* are treated as hyperparameters. At the start of each control episode, entries of the memory matrix $M_0$ are independently sampled from a unit normal distribution, $(M_0)_{ij} \sim \mathcal {N}(0.1)$. In order to compute the update at time-step *n*, we break down eq. (14) into two steps:

16 $$\begin{aligned} \bar{M}_{n}&= \text {Transformer}^* (M_{n-1}, o_n), \end{aligned}$$

17 $$\begin{aligned} M_n&= \bar{M}_{n} \, W_1 + M_{n-1} \, W_2. \end{aligned}$$

Here, we first apply a modified Transformer (to be described shortly) in eq. (16) to obtain the matrix $\bar{M}_{n}$. Then, we perform a learnable slow-moving memory-wise update in eq. (17). Here, $W_i = \sigma (\tilde{W}_i)$ are square $s \times s$ matrices with learnable parameters $\tilde{W}_i$ for $i \in \{ 1,2 \}$ and $\sigma (x) = (1+e^{-x})^{-1}$ is the Sigmoid function. The weights are initialised such that $W_i = \frac{1}{2} I_{s}$ for $i \in \{ 1,2 \}$, where $I_{s}$ is the *s*-dimensional identity matrix.

18 $$\begin{aligned} \tilde{M}_n&= \text {LayerNorm}(M_n), \end{aligned}$$

19 $$\begin{aligned} x_n&= \text {MLP}(o_n). \end{aligned}$$

**Fig. 4:**
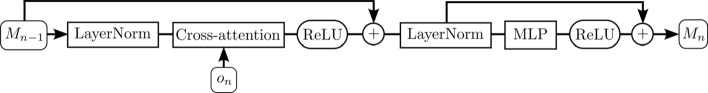
Overview of the modified Transformer architecture. The inputs are the previous memory $M_{n-1}$ and current observation $o_n$. The output is the updated memory $M_n$. The symbol ‘$+$’ stands for a residual connection, i.e. the map $(a,b) \mapsto a+b$. The symbol ‘MLP’ stands for multi-layer perceptron, while ‘ReLU’ stands for the ReLU activation function, i.e. the function $a \mapsto \max (0,a)$

The MLP is chosen such that its final dimension is *s*, so that $x_n \in \mathbb {R}^s$. Next, we append $x_n$ as the last row to $\tilde{M}_n$, forming the $(n+1) \times s$ matrix $T_n$. Then we compute:

20 $$\begin{aligned} Q_n = \tilde{M}_n W^q, \quad K_n = T_n W^k, \quad V_n = T_n W^v, \end{aligned}$$

21 $$\begin{aligned} S_n = \text {softmax} \left( \frac{Q_n \, K_n^T}{\sqrt{s}} \right) , \quad O_n = \tanh \left( S_n \, V_n \right) , \end{aligned}$$

where $W^q, W^k, W^v$ are learnable $s \times s$ matrices. Equation (20) is referred to as ‘cross-attention’, a simple modification to the original self-attention mechanism introduced in Vaswani et al. (2017). The $n \times s$ matrix $O_n$ is the output of the cross-attention layer and is mapped through residual connections, layer normalisation and MLPs as indicated in Fig. 4. We note that these subsequent layers act per-memory (i.e. row-wise on the matrix $O_n$). This completes the description of CHAROT.

## Results

We describe the details of training and introduce the performance metrics used to assess and compare different controller frameworks in Sect. 3.1. In Sect. 3.2 we then discuss when a KS environment is considered to be partially or fully observable. In the following Sects. 3.3 and 3.4 we then compare the performance of memory-based RL with memory-less RL.

### Training Details

We use TQC as a baseline RL algorithm for our experiments. The input of the TQC controller $\pi$ is then augmented with either the LSTM or CHAROT  framework as described in Sect. 2.4. The architectural modifications we make to the framework are however applicable to any actor-critic RL algorithm.

Training is carried out in an episodic manner, where each episode (i.e. numerical simulation of the KS equation) is $N=1000$ discrete timesteps, or $T=50$ physical time units long. We note carefully that a solution to the uncontrolled KS equation converges to the attractor around $t \approx 30$, see Fig. 3. After each episode, the parameters of the model are updated with *G* gradient updates using the ADAM optimiser, where the number of gradient updates equals the number of timesteps contained in the episode, $G=N$. Before training commences, we roll out 25 episodes where each action is taken randomly without updating the model parameters. This serves to increase the controller’s exploration of the action space. Each model is trained for a total number of 1000 episodes.

We assess the performance of a controller $\pi _\theta$ by periodically evaluating during training. During evaluation, the parameters $\theta$ of the controller are frozen and the controller acts deterministically. Each evaluation run produces a history $(h_n(x), \vec {a}_n)_{n=0, \dots , N}$ comprised of the state of the system $h_n(x) = h(t_n, x)$ and the action $\vec {a}_n = \vec {a}(t_n)$ taken by the agent at each discrete time step $t_n =n \, dt$. We then compute the discrete energy functional *E*, as defined in equation (4) periodically during training, to assess not only the final performance of the controller but also its speed of convergence. We evaluate every 2.5 episodes. Let us denote the energy obtained at the *k*-th evaluation run by $E_k, 1 \le k \le 40$. Thus, each training run of a particular RL framework produces a time series $E_k, 1 \le k \le 40$ of evaluation energies, which reflect the performance of the controller as a function of the number of training steps taken so far. To assess the final controller performance, we compute the metric

22 $$\begin{aligned} \mathcal {L} = \max _{k > 36} E_k. \end{aligned}$$

Note that the metric $\mathcal {L}$ measures the control loss after an initial training phase of 900 episodes, or $90 \%$ of the total training time. In order to increase the robustness of our results, we repeat each run several times, typically between five and ten.

The numerical experiments were carried out on the Edinburgh EIDF cluster, using NVIDIA A100 GPUs and on the Imperial College high performance computing service, using NVIDIA Quadro RTX 6000 s. Our code is implemented in the TorchRL package (Bou et al. 2023) and is available online. For the memoryless agent, training takes around 12 hours for 1000 episodes in total on a single GPU, whereas for both the LSTM and attention framework, training takes around 17 hours, see Table 2.

**Table 2:** Total wall-clock time for training an agent for $1 \times 10^6$ time steps using a single environment. The mean and standard deviation are computed across 10 repeated independent training runs

Architecture	Mean training time (hours)	Standard deviation (hours)
Memory-less	12.3	1.5
LSTM	17.0	1.8
CHAROT	17.5	0.9

### From Fully Observable to Partially Observable

**Fig. 5:**
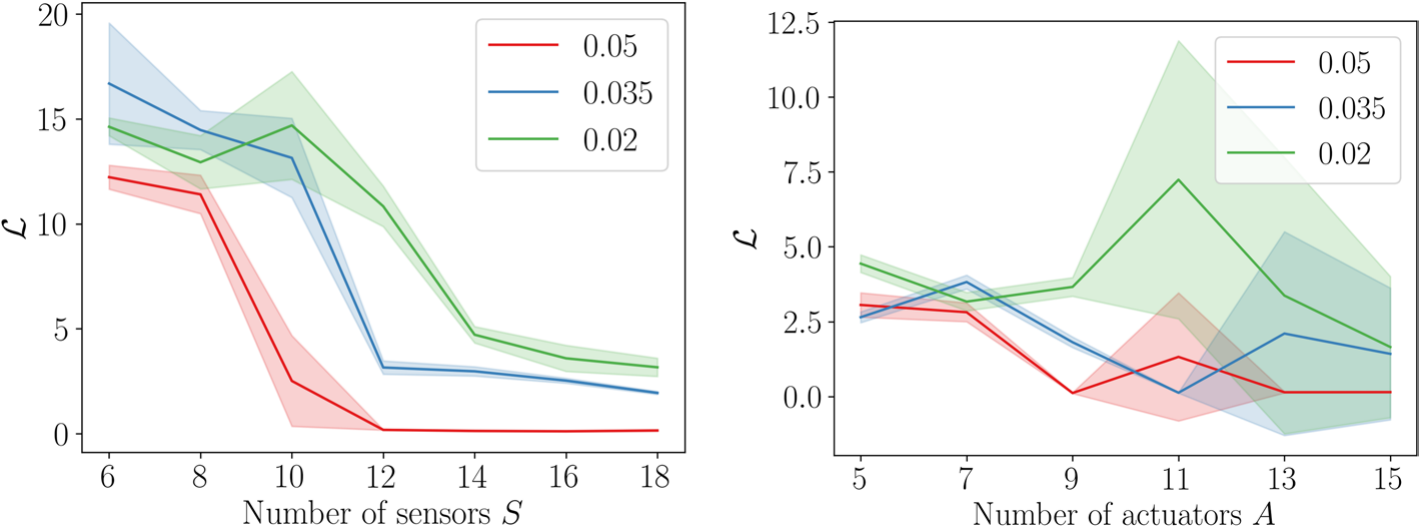
*Left*: Ablation study for the number of sensors *S* for four different values of $\nu$. We use a fixed value of $A=9$ actuators and trained the system for 500 episodes. Each training run was completed five times and the shaded areas represent the $95\%$ confidence interval of the control loss $\mathcal {L}$. *Right*: Ablation study for the number of actuators *A*. The number of sensors is fixed to $S=64$ (so that $o_t = h(t, \cdot )$). The run settings are identical to the left figure and the shaded areas again represent $95\%$ confidence intervals computed over five repeated runs

The difficulty of the KS control problem increases as the number of sensors *S* available to the controller decreases and the environment therefore becomes less observable. We train a memory-less RL controller on the KS environment with $\nu =0.05$ for different numbers of sensors. In this way, we quantify the drop in performance as the number of sensors decreases. Figure 5 summarises our key findings: As the number sensors *S* decreases, so does the performance of the memory-less controller, eventually leading to a breakdown of convergence.

The performance of the memory-less controller depends not only on the number of sensors *S*, but also the parameter $\nu$, the number of Gaussian actuators *A*, the width of the Gaussian curves which are used in the forcing term, the frequency at which the controller interacts with the environment, as well as the architectural details of the controller used. This is underlined by the results in Fig. 5, where we trained the memory-less RL controller on the fully observable KS environment ($o_n = u(t_n, \cdot )$) for different numbers of actuators *A*. Figure 5 shows that performance of a memory-less RL agent improves as the number of actuators available to the controller increases. Therefore, we may conclude that the control problem becomes easier if more actuators are available to the controller.

### Comparing Memory-Based RL with Memory-Less RL

**Fig. 6:**
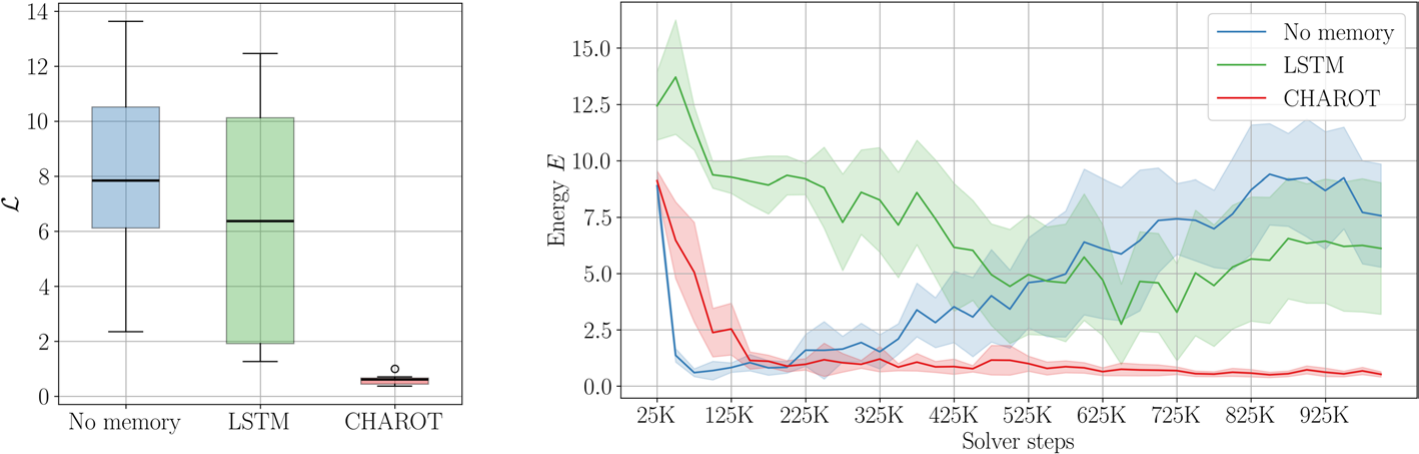
Comparison of the memory-less, LSTM, and attention-based controller for the KS environment with $\nu =0.05$, $S=10$ sensors and $A=9$ actuators. *Left*: Box plot of the performance metric $\mathcal {L}$ for ten repeated runs. *Right*: Energy $E_k$ as a function of the number of environment steps. The solid lines represent the mean across the repeated runs, while the shaded areas represent $95\%$ confidence intervals. Notice how the memory-less controller initially converges but subsequently unlearns catastrophically within a few training steps. Each framework is trained for a fixed number of 1000 episodes

**Fig. 7:**
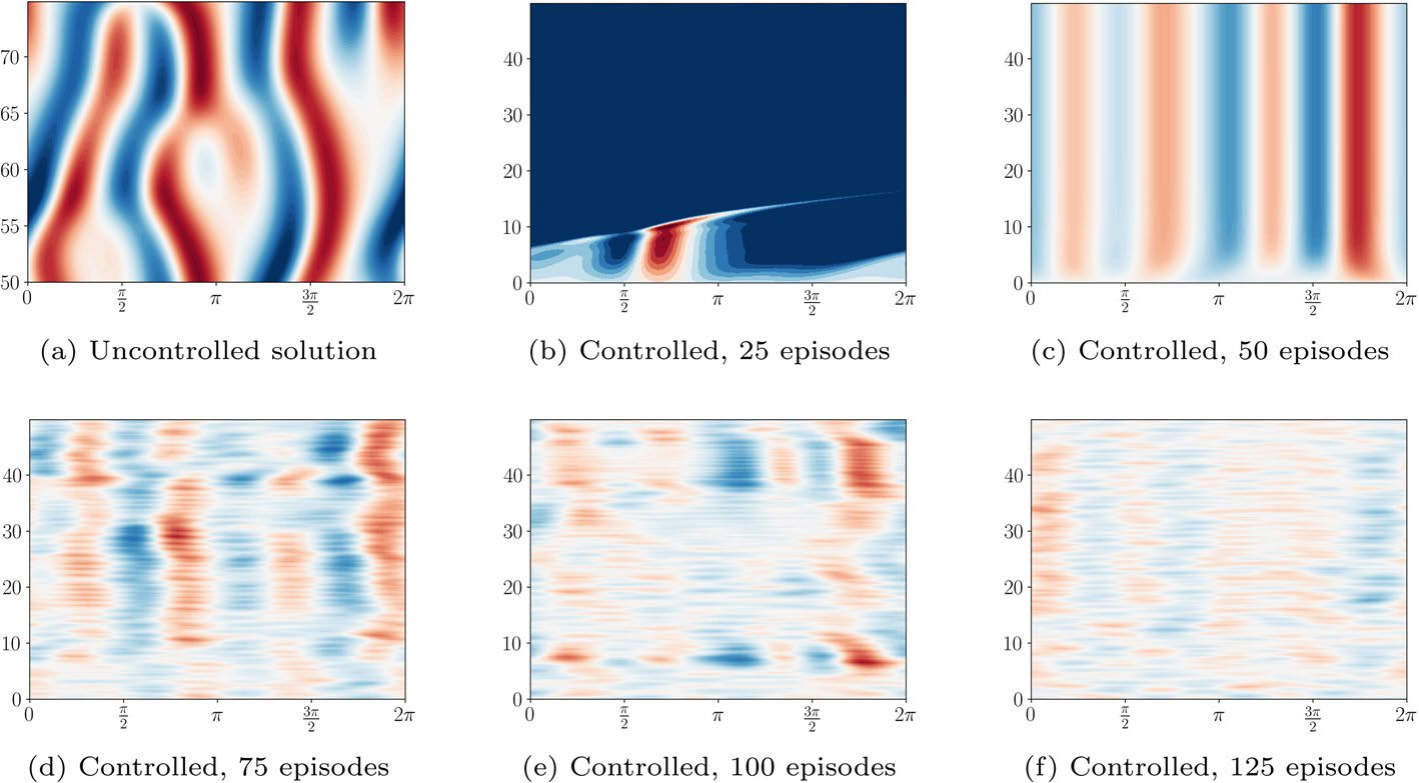
Evolution of an uncontrolled solution to the KS equation with $\nu =0.05$ (subfigure (a)) and the evaluation solution controlled by the attention-based controller evaluated after 25, 50, 75, 100, 125 episodes respectively (subfigures (b)-(f)). Initially, the controller does not manage to stabilise the solution around the trivial zero solution. Gradually, the controller becomes better at stabilising around the zero solution. After 125 episodes, the controller successfully stabilises the solution around the trivial zero solution. The evaluation plots for higher episode numbers look almost indistinguishable from the one for 125 episodes, so that we omit displaying them here

We perform RL using three different frameworks in a moderately chaotic regime. We evaluate the memory-less RL controller, the classical LSTM controller and the attention-based controller on the KS environment with $\nu =0.05$ and $S=10$ sensors and $A=9$ actuators. This ensures that the dynamics are moderately chaotic, see Sect. 2.2, and partially observable. We repeat each run ten times to obtain robust results. An example of the obtained evaluation solutions can be seen in Fig. 7.

The resulting box plot for the performance metric $\mathcal {L}$ and the curves $E_k$ are shown in Fig. 6. In Fig. 6 it can be seen that the attention-based controller outperforms both the controller without memory and the controller with LSTM memory within the given training budget of $10^6$ time steps. While we cannot rule out the possibility that the LSTM and memory-less frameworks might converge given an even larger number of training steps, we can conclude with certainty that the attention framework converges significantly faster and more robustly.

The controller augmented with LSTM only very slightly outperforms the memory-less controller. This is somewhat surprising given the prevalence of using LSTMs for solving POMDPs in the RL literature. The LSTM architecture presents convergence stiffness, which leads to a significantly slower convergence rate compared to the attention architecture. Discerning the precise reason for this drop in performance is a non-trivial endeavour, as understanding the internal workings of neural networks is complicated by their non-linear structures and the interactions between layers, which can obscure the relationships between inputs and outputs, making it challenging to pinpoint the exact factors influencing model behavior (Lipton 2018; Zhang et al. 2021). See also the discussion in Sect. 3.4.

Finally, the energy plot in Fig. 6 also shows that the memory-less controller initially learns quickly, but subsequently unlearns catastrophically, leading to a deterioration in performance. Catastrophic unlearning in RL agents is prevalent in dynamic environments, like partially observable Markov decision processes (POMDPs), where an agent must adapt to new states or observations while maintaining prior learning (McCloskey and Cohen 1989; Kaushik et al. 2021; Dick et al. 2020). This issue often indicates that the model’s learning strategy is overly focused on recent data, neglecting the value of previously acquired experiences. In our case, the addition of a attention-based memory module resolves this issue, while the addition of an LSTM-based memory does not, due to convergence stiffness and error accumulation (see also the discussion in the following paragraph).

### Performance in More Chaotic Regimes

**Fig. 8:**
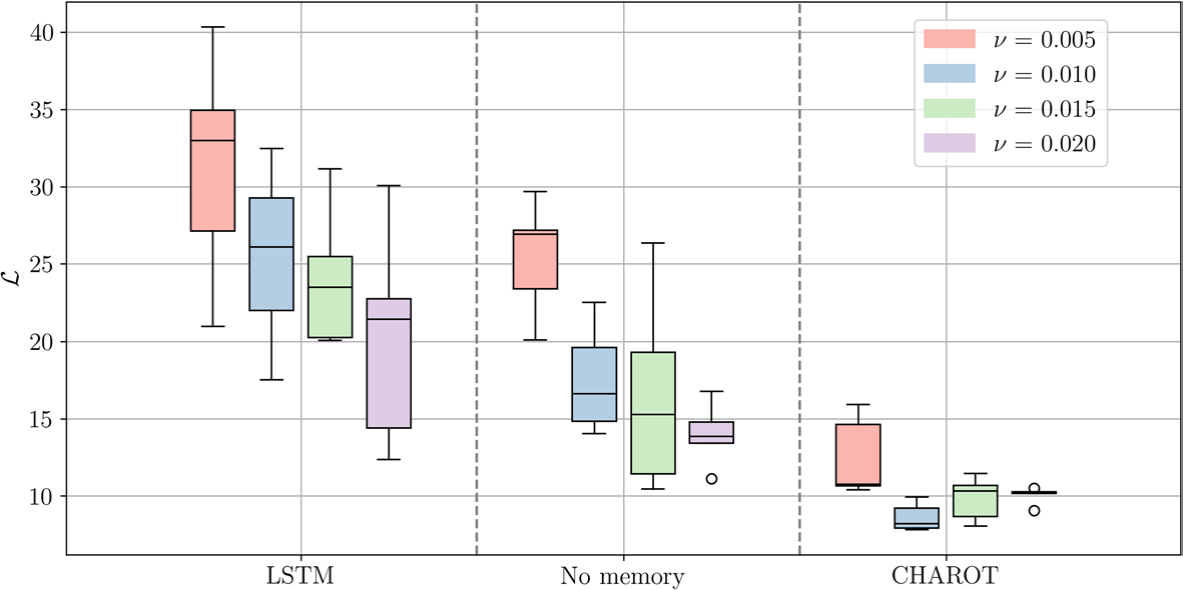
Box plots of the performance metric $\mathcal {L}$ across five repeated runs, computed for several small values of $\nu$. Notice that the KS environment becomes fully chaotic for $\nu \le 0.01$. CHAROT reliably outperforms both the LSTM framework and the memory-less controller. However, none of the algorithms fully converges to $\mathcal {L} \approx 0$ in the highly chaotic regime

In order to assess how well each of the three controllers generalises across different dynamic regimes, we trained the controllers for different values of $\nu$. We considered a range of $0.005 \le \nu \le 0.05$, which spans both the ‘weakly’ and ‘fully’ chaotic regime, see Table 1. More precisely, we chose the values $\nu = 0.005, 0.01, 0.015, 0.02$ since the system becomes fully chaotic at $\nu =0.01$. The chosen values therefore span the dynamic range both sides of the transition to chaos. For each value of $\nu$ considered, we train the three RL frameworks from scratch and then compute the metric $\mathcal {L}$ defined in equation (22). In order to obtain more robust results (while still using a manageable amount of compute), we repeat each experiment five times. The results are displayed in Fig. 8.

Figure 8 allows us to deduce that the attention-based framework generalises better to smaller values of $\nu$, and thus to more highly chaotic systems. We emphasize that re-training the frameworks in more chaotic domains enables us to assess the suitability of each framework for handling increasingly complex dynamics. Although exploring how well the trained models interpolate or extrapolate to unseen dynamical regimes is also an important direction, we leave this investigation for future work. While it is not trivial to discern the exact mechanism behind the superior performance of attention-based mechanisms, we note the following here: In chaotic systems, minuscule changes in the controller’s output can lead to large deviations in the system dynamics. When using an LSTM, these small errors accumulate quickly (since the output is computed in a strictly sequential manner). When using attention, all states are mapped through the model simultaneously, which makes it more robust to small changes or inaccuracies in controller input and output.

## Conclusion

In this study, we applied maximum-entropy reinforcement learning (TQC) to the problem of controlling the chaotic KS environment, while making only partial information about the state available to the controller. In order to keep our model as close to a real-world engineering scenario as possible, we only used one parallel environment during training, which reflects the conditions one might find in an experimental fluid dynamics use-case.

We characterised the drop in performance as the controller receives increasingly sparse information about the system state. We found that performance decreases as the number of sensors available to the controller decreases, and similarly, performance also decreases as the number of actuators available to the controller decreases.

We then compared the baseline TQC controller (without memory) to two different methods of converting the POMDP problem to a (fully observable) MDP problem: recurrent neural networks and the author’s novel attention-based memory framework, CHAROT. When comparing the performance on a mildly chaotic regime ($\nu = 0.05$), we found that the attention-based mechanism converges robustly, while the RNN-based framework and the memory-less controller fail to converge entirely. Furthermore, for highly chaotic regimes ($0.005 \le \nu \le 0.02$), we find that CHAROT  outperforms the alternatives by approximately $200 \%$. In the more highly chaotic regimes however, none of the frameworks successfully manages to stabilise the trivial zero solution fully, highlighting the difficulty of stabilising the state of a chaotic system.

It was shown that the prevailing approach of converting POMDPs to MDPs (using RNNs) may fail in scenarios when an attention-based memory framework succeeds in stabilising the system. Therefore, we suggest that attention-based architectures, such as the one implemented in this work, may be more successful when controlling or stabilising chaotic flows. In particular, attention-based memory may prove to be a more adaptable framework for manipulating 3D fluid flows to achieve outcomes such as drag reduction or increased energy efficiency. By leveraging attention-based architectures, it is possible to develop more robust and effective controllers that can operate successfully in realistic and complex fluid dynamic environments.

## Data Availability

The authors declare that the data supporting the findings of this study are available within the paper and its supplementary information files. The source code used to generate all datasets is publicly available at https://github.com/maxweissenbacher/charot.

## References

[CR1] Ashinsky, G.S.: Nonlinear analysis of hydrodynamic instability in laminar flames–i. derivation of basic equations. In: Dynamics of Curved Fronts, pp. 459–488. Elsevier, ??? (1988)

[CR2] Beaudoin, J.-F., Cadot, O., Aider, J.-L., Wesfreid, J.-E.: Drag reduction of a bluff body using adaptive control methods. Phys. Fluids (2006). 10.1063/1.2236305

[CR3] Bengio, Y., Simard, P., Frasconi, P.: Learning long-term dependencies with gradient descent is difficult. IEEE Trans. Neural Netw. **5**(2), 157–166 (1994) 18267787 10.1109/72.279181

[CR4] Blatter, G., Feigel’man, M.V., Geshkenbein, V.B., Larkin, A.I., Vinokur, V.M.: Vortices in high-temperature superconductors. Rev. Mod. Phys. **66**(4), 1125 (1994)

[CR5] Bou, A., Bettini, M., Dittert, S., Kumar, V., Sodhani, S., Yang, X., Fabritiis, G.D., Moens, V.: TorchRL: A data-driven decision-making library for PyTorch (2023)

[CR6] Brackston, R.D., De La Cruz, J.G., Wynn, A., Rigas, G., Morrison, J.: Stochastic modelling and feedback control of bistability in a turbulent bluff body wake. J. Fluid Mech. **802**, 726–749 (2016)

[CR7] Brown, T., Mann, B., Ryder, N., Subbiah, M., Kaplan, J.D., Dhariwal, P., Neelakantan, A., Shyam, P., Sastry, G., Askell, A., et al.: Language models are few-shot learners. Adv. Neural. Inf. Process. Syst. **33**, 1877–1901 (2020)

[CR8] Bucci, M.A., Semeraro, O., Allauzen, A., Wisniewski, G., Cordier, L., Mathelin, L.: Control of chaotic systems by deep reinforcement learning. Proc. Royal Soc. A **475**(2231), 20190351 (2019) 10.1098/rspa.2019.0351 PMC6894543 31824214

[CR9] Cassandra, A.R., Kaelbling, L.P., Littman, M.L.: Acting optimally in partially observable stochastic domains. In: Aaai, 94, 1023–1028 (1994)

[CR10] Chatzimanolakis, M., Weber, P., Koumoutsakos, P.: Learning in two dimensions and controlling in three: generalizable drag reduction strategies for flows past circular cylinders through deep reinforcement learning. Phys. Rev. Fluids **9**(4), 043902 (2024)

[CR11] Collet, P., Eckmann, J.-P., Epstein, H., Stubbe, J.: Analyticity for the kuramoto-sivashinsky equation. Physica D **67**(4), 321–326 (1993)

[CR12] Collet, P., Eckmann, J.-P., Epstein, H., Stubbe, J.: A global attracting set for the kuramoto-sivashinsky equation (1993)

[CR13] Conte, R., Musette, M.: Painleve analysis and backlund transformation in the kuramoto-sivashinsky equation. J. Phys. A: Math. Gen. **22**(2), 169 (1989)

[CR14] Cvitanović, P., Davidchack, R.L., Siminos, E.: On the state space geometry of the kuramoto-sivashinsky flow in a periodic domain. SIAM J. Appl. Dyn. Syst. **9**(1), 1–33 (2010)

[CR15] Dankowicz, H., Holmes, P., Berkooz, G., Elezgaray, J.: Local models of spatio-temporally complex fields. Physica D **90**(4), 387–407 (1996)

[CR16] Devlin, J., Chang, M.-W., Lee, K., Toutanova, K.: Bert: Pre-training of deep bidirectional transformers for language understanding. arXiv preprint arXiv:1810.04805 (2018)

[CR17] Dey, R., Salem, F.M.: Gate-variants of gated recurrent unit (gru) neural networks. In: 2017 IEEE 60th International Midwest Symposium on Circuits and Systems (MWSCAS), pp. 1597–1600 (2017). IEEE

[CR18] Dick, J., Ladosz, P., Ben-Iwhiwhu, E., Shimadzu, H., Kinnell, P., Pilly, P.K., Kolouri, S., Soltoggio, A.: Detecting changes and avoiding catastrophic forgetting in dynamic partially observable environments. Front. Neurorobot. **14**, 578675 (2020) 33424575 10.3389/fnbot.2020.578675 PMC7787001

[CR19] Dodge, Y.: The oxford dictionary of statistical terms. Oxford University Press, USA (2003)

[CR20] Dosovitskiy, A., Beyer, L., Kolesnikov, A., Weissenborn, D., Zhai, X., Unterthiner, T., Dehghani, M., Minderer, M., Heigold, G., Gelly, S., et al.: An image is worth 16x16 words: Transformers for image recognition at scale. arXiv preprint arXiv:2010.11929 (2020)

[CR21] Edson, R.A., Bunder, J.E., Mattner, T.W., Roberts, A.J.: Lyapunov exponents of the kuramoto-sivashinsky pde. ANZIAM J. **61**(3), 270–285 (2019)

[CR22] Espeholt, L., Soyer, H., Munos, R., Simonyan, K., Mnih, V., Ward, T., Doron, Y., Firoiu, V., Harley, T., Dunning, I., et al. Impala: Scalable distributed deep-rl with importance weighted actor-learner architectures. In: International Conference on Machine Learning, pp. 1407–1416 (2018). PMLR

[CR23] Fisher, D.S., Fisher, M.P., Huse, D.A.: Thermal fluctuations, quenched disorder, phase transitions, and transport in type-ii superconductors. Phys. Rev. B **43**(1), 130 (1991) 10.1103/physrevb.43.130 9996198

[CR24] Foias, C., Nicolaenko, B., Sell, G.R., Temam, R.: Inertial manifolds for the kuramoto-sivashinsky equation and an estimate of their lowest dimension (1986)

[CR25] Giacomelli, L., Otto, F.: New bounds for the kuramoto-sivashinsky equation. Commun. Pure Appl. Mathe. J. Issued Courant Instit. Mathe. Sci. **58**(3), 297–318 (2005)

[CR26] Giannenas, A.E., Laizet, S., Rigas, G.: Harmonic forcing of a laminar bluff body wake with rear pitching flaps. J. Fluid Mech. **945**, 5 (2022)

[CR27] Greff, K., Srivastava, R.K., Koutník, J., Steunebrink, B.R., Schmidhuber, J.: Lstm: A search space odyssey. IEEE Transa. Neural Netw. Learn. Syst. **28**(10), 2222–2232 (2016) 10.1109/TNNLS.2016.2582924 27411231

[CR28] Haarnoja, T., Zhou, A., Abbeel, P., Levine, S.: Soft actor-critic: Off-policy maximum entropy deep reinforcement learning with a stochastic actor. In: International Conference on Machine Learning, pp. 1861–1870 (2018). PMLR

[CR29] Hausknecht, M., Stone, P.: Deep recurrent q-learning for partially observable mdps. In: 2015 Aaai Fall Symposium Series (2015)

[CR30] Heess, N., Wayne, G., Silver, D., Lillicrap, T., Erez, T., Tassa, Y.: Learning continuous control policies by stochastic value gradients. Advances in neural information processing systems **28** (2015)

[CR31] Hochreiter, S., Schmidhuber, J.: Long short-term memory. Neural Comput. **9**(8), 1735–1780 (1997) 9377276 10.1162/neco.1997.9.8.1735

[CR32] Holmes, P.: Turbulence, coherent structures, dynamical systems and symmetry. Cambridge university press (2012)

[CR33] Horgan, D., Quan, J., Budden, D., Barth-Maron, G., Hessel, M., Van Hasselt, H., Silver, D.: Distributed prioritized experience replay. arXiv preprint arXiv:1803.00933 (2018)

[CR34] Jeon, J., Rabault, J., Vasanth, J., Alcántara-Ávila, F., Baral, S., Vinuesa, R.: Advanced deep-reinforcement-learning methods for flow control: group-invariant and positional-encoding networks improve learning speed and quality. arXiv preprint arXiv:2407.17822 (2024)

[CR35] Kaelbling, L.P., Littman, M.L., Cassandra, A.R.: Planning and acting in partially observable stochastic domains. Artif. Intell. **101**(1–2), 99–134 (1998)

[CR36] Kalogirou, A., Keaveny, E.E., Papageorgiou, D.T.: An in-depth numerical study of the two-dimensional kuramoto-sivashinsky equation. Proc. Royal Soc. A: Mathe. Phys. Eng. Sci. **471**(2179), 20140932 (2015) 10.1098/rspa.2014.0932 PMC4528647 26345218

[CR37] Kaplan, J.L., Yorke, J.A. 2006 Chaotic behavior of multidimensional difference equations. Functional Differential Equations and Approximation of Fixed Points: Proceedings. 1978, 204–227

[CR38] Kaushik, P., Gain, A., Kortylewski, A., Yuille, A.: Understanding catastrophic forgetting and remembering in continual learning with optimal relevance mapping. arXiv preprint arXiv:2102.11343 (2021)

[CR39] Kuramoto, Y.: Diffusion-induced chaos in reaction systems. Prog. Theor. Phys. Suppl. **64**, 346–367 (1978)

[CR40] Kuramoto, Y., Tsuzuki, T.: Persistent propagation of concentration waves in dissipative media far from thermal equilibrium. Progress Theoret. Phys. **55**(2), 356–369 (1976)

[CR41] Kuznetsov, A., Shvechikov, P., Grishin, A., Vetrov, D.: Controlling overestimation bias with truncated mixture of continuous distributional quantile critics. In: International Conference on Machine Learning, pp. 5556–5566 (2020). PMLR

[CR42] Lillicrap, T.P., Hunt, J.J., Pritzel, A., Heess, N., Erez, T., Tassa, Y., Silver, D., Wierstra, D.: Continuous control with deep reinforcement learning. arXiv preprint arXiv:1509.02971 (2015)

[CR43] Linot, A.J., Graham, M.D.: Deep learning to discover and predict dynamics on an inertial manifold. Phys. Rev. E **101**(6), 062209 (2020) 32688613 10.1103/PhysRevE.101.062209

[CR44] Lipton, Z.C.: The mythos of model interpretability: In machine learning, the concept of interpretability is both important and slippery. Queue **16**(3), 31–57 (2018)

[CR45] Lorenz, E.N.: Deterministic nonperiodic flow. J. Atmos. Sci. **20**(2), 130–141 (1963)

[CR46] McCloskey, M., Cohen, N.J.: Catastrophic interference in connectionist networks: The sequential learning problem **24**, 109–165 (1989)

[CR47] Mnih, V., Badia, A.P., Mirza, M., Graves, A., Lillicrap, T., Harley, T., Silver, D., Kavukcuoglu, K.: Asynchronous methods for deep reinforcement learning. In: International Conference on Machine Learning, pp. 1928–1937 (2016). PMLR

[CR48] Mnih, V., Kavukcuoglu, K., Silver, D., Graves, A., Antonoglou, I., Wierstra, D., Riedmiller, M.: Playing atari with deep reinforcement learning. arXiv preprint arXiv:1312.5602 (2013)

[CR49] Morad, S., Kortvelesy, R., Bettini, M., Liwicki, S., Prorok, A.: Popgym: Benchmarking partially observable reinforcement learning. arXiv preprint arXiv:2303.01859 (2023)

[CR50] Ni, T., Eysenbach, B., Salakhutdinov, R.: Recurrent model-free rl can be a strong baseline for many pomdps. arXiv preprint arXiv:2110.05038 (2021)

[CR51] Nicolaenko, B., Scheurer, B., Temam, R.: Some global dynamical properties of the kuramoto-sivashinsky equations: nonlinear stability and attractors. Physica D **16**(2), 155–183 (1985)

[CR52] Otto, F.: Optimal bounds on the kuramoto-sivashinsky equation. J. Funct. Anal. **257**(7), 2188–2245 (2009)

[CR53] Özalp, E., Margazoglou, G., Magri, L.: Physics-informed long short-term memory for forecasting and reconstruction of chaos. arXiv preprint arXiv:2302.10779 (2023) 10.1063/5.0159479 37671991

[CR54] Palmer, T.N.: Extended-range atmospheric prediction and the lorenz model. Bull. Am. Meteor. Soc. **74**(1), 49–66 (1993)

[CR55] Papageorgiou, D.T., Smyrlis, Y.S.: The route to chaos for the kuramoto-sivashinsky equation. Theoret. Comput. Fluid Dyn. **3**(1), 15–42 (1991)

[CR56] Paris, R., Beneddine, S., Dandois, J.: Robust flow control and optimal sensor placement using deep reinforcement learning. J. Fluid Mech. **913**, 25 (2021)

[CR57] Parisotto, E., Song, F., Rae, J., Pascanu, R., Gulcehre, C., Jayakumar, S., Jaderberg, M., Kaufman, R.L., Clark, A., Noury, S., et al. Stabilizing transformers for reinforcement learning. In: International Conference on Machine Learning, pp. 7487–7498 (2020). PMLR

[CR58] Pascanu, R.: On the difficulty of training recurrent neural networks. arXiv preprint arXiv:1211.5063 (2013)

[CR59] Pathak, J., Lu, Z., Hunt, B.R., Girvan, M., Ott, E.: Using machine learning to replicate chaotic attractors and calculate lyapunov exponents from data. Chaos: Interdiscip. J. Nonlinear Sci. (2017). 10.1063/1.5010300 10.1063/1.5010300 29289043

[CR60] Peng, X.B., Andrychowicz, M., Zaremba, W., Abbeel, P.: Sim-to-real transfer of robotic control with dynamics randomization. In: 2018 IEEE International Conference on Robotics and Automation (ICRA), pp. 3803–3810 (2018). IEEE

[CR61] Pritzel, A., Uria, B., Srinivasan, S., Badia, A.P., Vinyals, O., Hassabis, D., Wierstra, D., Blundell, C.: Neural episodic control. In: International Conference on Machine Learning, pp. 2827–2836 (2017). PMLR

[CR62] Rabault, J., Kuchta, M., Jensen, A., Réglade, U., Cerardi, N.: Artificial neural networks trained through deep reinforcement learning discover control strategies for active flow control. J. Fluid Mech. **865**, 281–302 (2019)

[CR63] Rabault, J., Kuhnle, A.: Accelerating deep reinforcement learning strategies of flow control through a multi-environment approach. Phys. Fluids (2019). 10.1063/1.5116415

[CR64] Radford, A., Wu, J., Child, R., Luan, D., Amodei, D., Sutskever, I., et al.: Language models are unsupervised multitask learners. OpenAI blog **1**(8), 9 (2019)

[CR65] Rathnasingham, R., Breuer, K.S.: Active control of turbulent boundary layers. J. Fluid Mech. **495**, 209–233 (2003)

[CR66] Santoro, A., Faulkner, R., Raposo, D., Rae, J., Chrzanowski, M., Weber, T., Wierstra, D., Vinyals, O., Pascanu, R., Lillicrap, T.: Relational recurrent neural networks. Advances in neural information processing systems **31** (2018)

[CR67] Sivashinsky, G.I.: On flame propagation under conditions of stoichiometry. SIAM J. Appl. Math. **39**(1), 67–82 (1980)

[CR68] Sondik, E.J.: The optimal control of partially observable markov processes over the infinite horizon: Discounted costs. Oper. Res. **26**(2), 282–304 (1978)

[CR69] Sutton, R.S., Barto, A.G.: Reinforcement learning: An introduction. MIT press (2018)

[CR70] Tadmor, E.: The well-posedness of the kuramoto-sivashinsky equation. SIAM J. Math. Anal. **17**(4), 884–893 (1986)

[CR71] Takens, F.: Detecting strange attractors in turbulence. In: Dynamical Systems and Turbulence, Warwick 1980: Proceedings of a Symposium Held at the University of Warwick 1979/80, pp. 366–381 (2006). Springer

[CR72] Temam, R., Wang, X.M.: Estimates on the lowest dimension of inertial manifolds for the Kuramoto-Sivashinsky equation in the general case. Differ. Integral Eq. **7**(3–4), 1095–1108 (1994)

[CR73] Temam, R.: Infinite-dimensional dynamical systems in mechanics and physics. Springer Science & Business Media **68** (2012)

[CR74] Vasanth, J., Rabault, J., Alcántara-Ávila, F., Mortensen, M., Vinuesa, R.: Multi-agent reinforcement learning for the control of three-dimensional rayleigh-b’enard convection. arXiv preprint arXiv:2407.21565 (2024) 10.1007/s10494-024-00619-2 PMC12507992 41079146

[CR75] Vaswani, A., Shazeer, N., Parmar, N., Uszkoreit, J., Jones, L., Gomez, A.N., Kaiser, Ł., Polosukhin, I.: Attention is all you need. Advances in neural information processing systems **30** (2017)

[CR76] Vignon, C., Rabault, J., Vinuesa, R.: Recent advances in applying deep reinforcement learning for flow control: Perspectives and future directions. Phys. Fluids (2023). 10.1063/5.0143913

[CR77] Wang, Q., Yan, L., Hu, G., Chen, W., Rabault, J., Noack, B.R.: Dynamic feature-based deep reinforcement learning for flow control of circular cylinder with sparse surface pressure sensing. J. Fluid Mech. **988**, 4 (2024)

[CR78] Weissenbacher, M., Borovykh, A., Rigas, G.: CHAROT: Robustly controlling chaotic PDEs with partial observations. In: ICLR 2024 Workshop on AI4DifferentialEquations In Science (2024). https://openreview.net/forum?id=SytuCWihJr

[CR79] Wen, Q., Zhou, T., Zhang, C., Chen, W., Ma, Z., Yan, J., Sun, L.: Transformers in time series: A survey. arXiv preprint arXiv:2202.07125 (2022)

[CR80] Wijmans, E., Savva, M., Essa, I., Lee, S., Morcos, A.S., Batra, D.: Emergence of maps in the memories of blind navigation agents. arXiv preprint arXiv:2301.13261 (2023)

[CR81] Wittenberg, R.W., Holmes, P.: Scale and space localization in the kuramoto-sivashinsky equation. Chaos: Interdiscip. J. Nonlinear Sci. **9**(2), 452–465 (1999) 10.1063/1.166419 12779842

[CR82] Xia, C., Zhang, J., Kerrigan, E.C., Rigas, G.: Active flow control for bluff body drag reduction using reinforcement learning with partial measurements. J. Fluid Mech. **981**, 17 (2024)

[CR83] Xu, D., Zhang, M.: Reinforcement-learning-based control of convectively unstable flows. J. Fluid Mech. **954**, 37 (2023)

[CR84] Yu, H., Bertsekas, D.P.: On near optimality of the set of finite-state controllers for average cost pomdp. Math. Oper. Res. **33**(1), 1–11 (2008)

[CR85] Yu, Y., Si, X., Hu, C., Zhang, J.: A review of recurrent neural networks: Lstm cells and network architectures. Neural Comput. **31**(7), 1235–1270 (2019) 31113301 10.1162/neco_a_01199

[CR86] Zeng, K., Graham, M.D.: Symmetry reduction for deep reinforcement learning active control of chaotic spatiotemporal dynamics. Phys. Rev. E **104**(1), 014210 (2021) 34412246 10.1103/PhysRevE.104.014210

[CR87] Zeng, K., Linot, A.J., Graham, M.D.: Data-driven control of spatiotemporal chaos with reduced-order neural ode-based models and reinforcement learning. Proc. Royal Soc. A **478**(2267), 20220297 (2022)

[CR88] Zhang, C., Bengio, S., Hardt, M., Recht, B., Vinyals, O.: Understanding deep learning (still) requires rethinking generalization. Commun. ACM **64**(3), 107–115 (2021)

[CR89] Zhu, Y., Mottaghi, R., Kolve, E., Lim, J.J., Gupta, A., Fei-Fei, L., Farhadi, A.: Target-driven visual navigation in indoor scenes using deep reinforcement learning. In: 2017 IEEE International Conference on Robotics and Automation (ICRA), pp. 3357–3364 (2017). IEEE

